# *Sarcophaga (Liosarcophaga) dux* (Diptera: Sarcophagidae): A flesh fly species of medical importance

**DOI:** 10.1186/0717-6287-47-14

**Published:** 2014-04-01

**Authors:** Kabkaew L Sukontason, Sangob Sanit, Tunwadee Klong-klaew, Jeffery K Tomberlin, Kom Sukontason

**Affiliations:** Department of Parasitology, Faculty of Medicine, Chiang Mai University, Chiang Mai, 50200 Thailand; Department of Entomology, Texas A&M University, College Station, TX USA

**Keywords:** *Sarcophaga dux*, Review literature, Thailand, Forensic entomology, Myiasis, Morphology, Adult, Immature stages

## Abstract

**Background:**

Although tropical climate of Thailand is suitably endowed with biodiversity of insects, flies of medical importance is not well investigated. Using information from literature search, fly survey approach and specialist’s experience, we review database of Sarcophaga (Liosarcophaga) dux Thomson (Diptera: Sarcophagidae), one of the priorities flesh fly species of medical importance in Thailand.

**Results:**

This review deals with morphology, bionomics and medical involvement. Important morphological characteristics of egg, larva, puparia and adult were highlighted with illustration and/or micrographs. Search pertaining to molecular analysis used for fly identification and developmental rate of larvae were included. Medical involvement of larvae was not only myiasis-producing agent in humans and animals, but associated with human death investigations.

**Conclusions:**

This information will enable us to accurate identify this species and to emphasis the increase medically important scene in Thailand.

## Background

*Sarcophaga (Liosarcophaga) dux* Thomson (= *exuberan* Pandellé) is a flesh fly (Diptera: Sarcophagidae) species of medical importance in many parts of the world [1]. Geographically, this fly prevails in many part of the world including, but not limited to, southern Europe [France] [2]; Oriental region [e.g., Thailand [3], Malaysia [4], India [5], Nepal [6], Saudi Arabia [7], Egypt [8], Myanmar, Philippines, Indonesia, Japan, Korea, Sri Lanka, Taiwan, China [6]; Australia; and Hawaii, USA [6]. This species is of medical importance as a myiasis-producing agent [9] as well as forensics as it is known to colonize decomposing human remains [1]. This paper reviews its adult morphology, bionomics and medical involvement.

## Results and discussion

### Morphology

Based on the fact that numerous flesh flies species exist in Thailand, information pertaining to morphology of flesh flies is significant for comparison into group and/or species level, particularly those of medical and forensic importance. Gathering information of all stages in *S. dux*’s life cycle would enable identification of this organism, leading to be applied in the primary step of forensic investigation. Much of the morphological traits come from LM and SEM observations.

#### Morphology of adults

**Figure 1:**
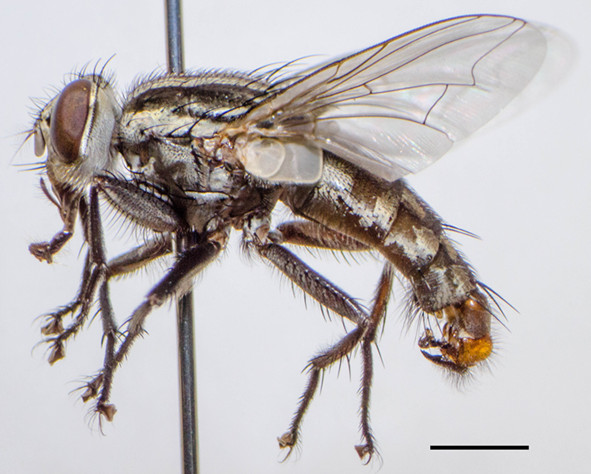
**Adult male of** ***S. dux*** **.** Online figure in color. Bar = 0.2 mm.

**Figure 2:**
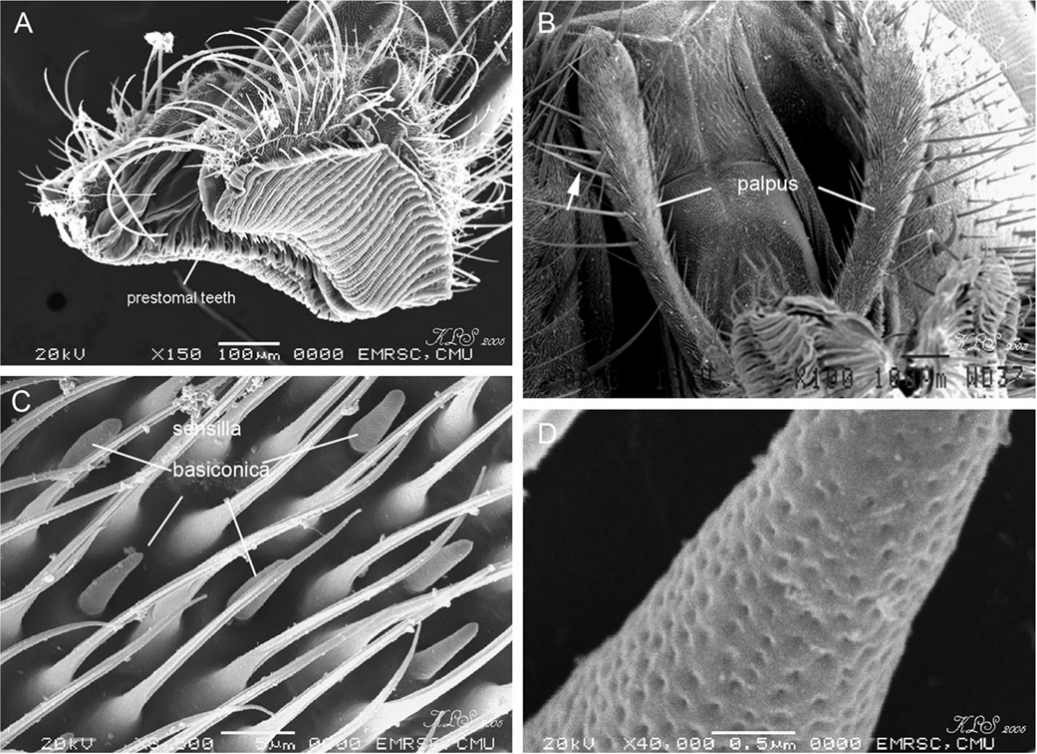
**Scanning electron micrographs of adult** ***S. dux*** **. A**: Sponging mouthpart showing everted labella lobes and numerous sensilla trichodea. Prestomal teeth are bifurcation at the tips. **B**: Palpus showing strong and long sensilla chaetica (arrow). **C**: Surface of palpus with amount of sensilla basiconica. **D**: Higher magnification of sensilla basiconica at palpus with perforation surface.

Adults are dull gray with three longitudinal black strips on the mesonotum, while the abdomen possess checkered or spotted pattern (Figure 1). Body length of male *S. dux* is medium to large size (7–12 mm). Important morphological characteristics used for differentiated this species from other forensically important flesh flies are follows: third antennal segment fuscous black, palpus entirely blackish, Postsutural *ac* present, genital segment 2 blackish or sometimes yellowish orange [10]. Notable features are located on the head including large compound eyes, antennae and a sponging mouthpart with prominent palps. To evaluate the number of ommatidia of medically important flies in Thailand, we designed a study by soaking the head in 20% potassium hydroxide solution in room temperature for 7 days, then the compound eye was dissected into six small parts which was placed onto a glass slide and flatted using a coverslip. Image of each part was manually counted by those printed part obtained using microscope from the computer [11]. Using this procedure, the average number of ommatidia in male compound eye was 6,032 ± 385 (left eye) and 6,032 ± 408 (right eye); whereas females had 6,073 ± 207 (left eye) and 6,100 ± 220 (right eye). Such great number of ommatidia would allow flies to perceive better visual resolution, and thereby implication for visual efficiency [12]. Our observation on the antennal sensilla using SEM revealed that antennal segment of both sex are endowed with several types of sensilla – sensilla chaetica, sensilla trichodea, sensilla basiconica, sensilla styloconica, sensilla coeloconica and sensory pits [13], allowing helpful in the perception various receptions (e.g., chemoreception, mechanoreception, olfactory). Similarly to antenna that endows several sensillae, our result displayed that sponging mouthpart of *S. dux* possess sensilla – sensilla trichodea, sensilla basiconica at labellar lobes; sensilla chaetica and sensilla basiconica at palpus (Figures 2A-D). The prestomal teeth are bifurcation at the tips (Figure 2A). Such features of sensilla observed over antenna, mouthpart and bifurcated prestomal teeth of *S. dux* were morphologically similar to previous published works of blow flies [12, 13].

**Figure 3:**
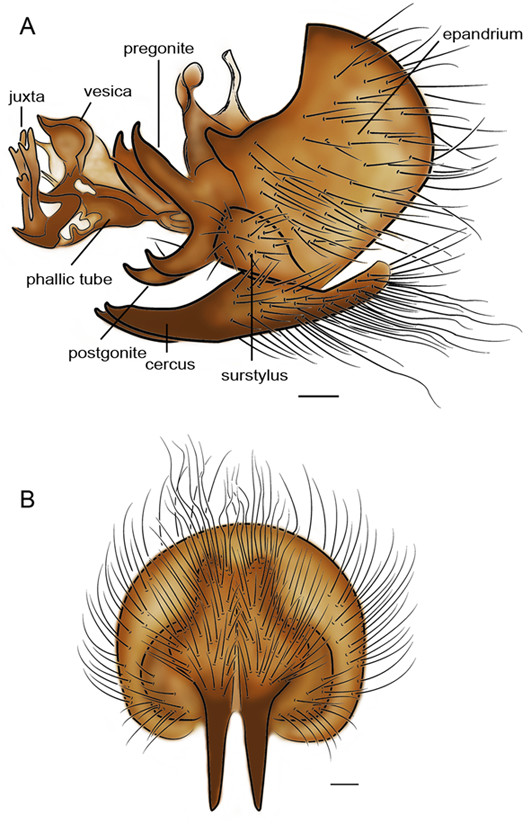
**Illustrations of male genitalia** ***S. dux*** **. A**: lateral view of genitalia displaying short, broad phallus, slightly curved upward apically of pregonite and postgonite, pointed and curved apically cerci and apically bifurcated juxta. **B**: Epandrium, cerci and surstyli, posterior. Online figure in color. Bar = 0.1 mm. Terminology followed Giroux et al. [15].

**Figure 4:**
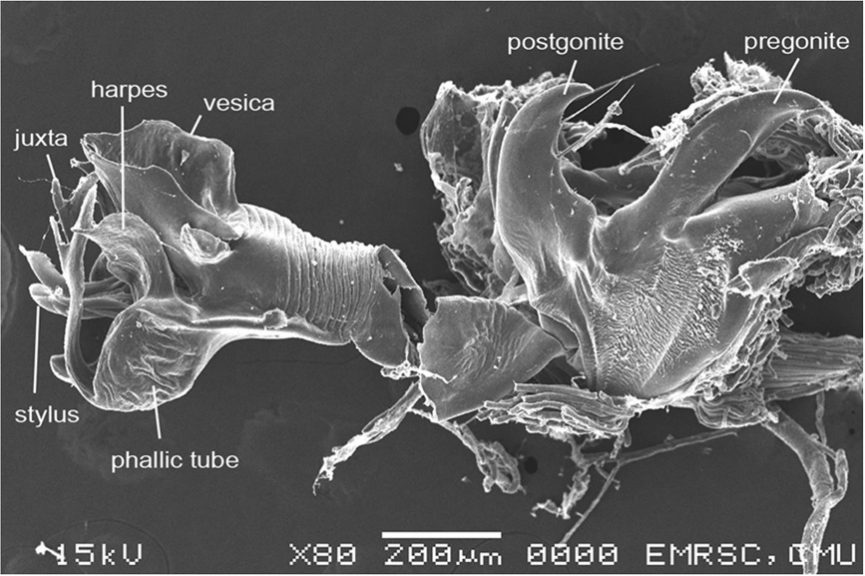
**Scanning electron micrograph of male genitalia** ***S. dux*** **showing trumpet-shaped, anteroventrally expanded vesica, bifurcated juxta.** Terminology followed Giroux et al. [15].

**Figure 5:**
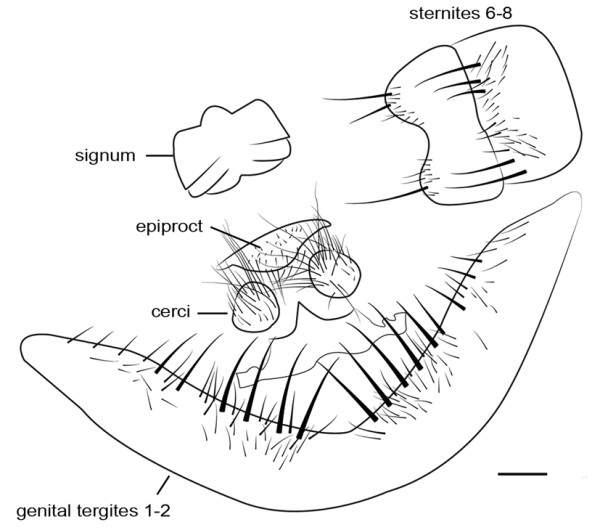
**Illustration of ovipositor** ***S. dux*** **.** Bar = 0.1 mm. Terminology followed Richet et al. [2].

As one of the three priorities of flesh fly species of medical importance in Thailand, characteristic of adult for identification was of emphasis. For morphological investigations, research in the form of drawings, LM and SEM images are still needed. Characteristics of male genitalia of *S. dux* have been displayed using SEM by Chaiwong et al. [14]. Based on the terminology of Giroux et al. [15], the phallus is a short, broad structure that is formed by a tubular base connected to a trumpet-shaped, anteroventrally expanded vesica. The juxta is apically bifurcated (Figures 3 and 4). The pregonite and postgonite are slightly curved upward apically. The cerci are pointed and curved apically. Male genitalia *S. dux* was distinctively feature and much different from several flesh fly species observed with SEM [15]. Such information obtained from either LM or SEM provide relatively ease for identification into species. As for females, the ovipositor is shown in Figure 5. Kurahashi and Chaiwong [16] provides most recent taxonomic key checklists of male flesh flies in Thailand to include *S. dux*.

**Figure 6:**
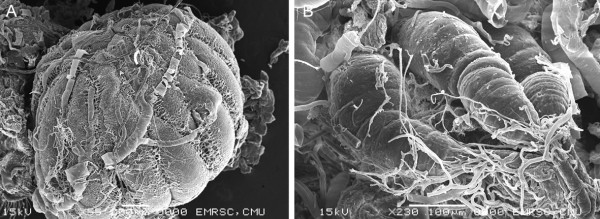
**Scanning electron micrographs of 7-d-old female** ***S. dux*** **. A**: Ovary. **B**: Spermathecae.

**Figure 7:**
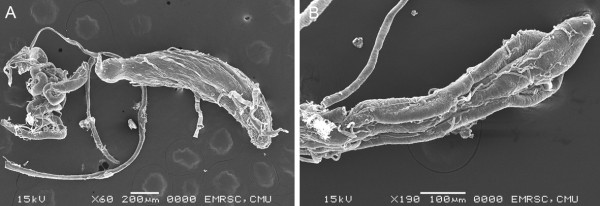
**Scanning electron micrographs of male** ***S. dux*** **. A**: testis and accessory gland. **B**: Higher magnification of distal part of accessory gland.

Information pertaining to morphology of internal organs of *S. dux* was very limited. Our dissection of reproductive organ in 7-d old female showed large ovaries, covered by an ovarian envelope comprising numerous ovariole which develop to be eggs with embryo inside (Figure 6A). The spermathecae has 3 lobes each being tubular in form (Figure 6B), of which this feature was distinctive with the globular structure that has been observed in blow fly, *Chrysomya megacephala* (F.) [17]. A pair of testis is elongated shape is present (Figure 7A). Accessory glands are tubular and proximally convoluted. The distal part of the gland form two distinct parts – long tubular structure lay above the long patch (Figure 7B).

#### Morphology of immature stages

*S. dux* can be oviparous. A morphological description of *S. dux* eggs was provided by Sukontason et al. [18]. They are elongated and slightly bean-shaped, measuring ~1.5 mm in length. The eggshell comprised of polygonal patterns externally, and sectioning displayed multiple layers of the eggshell: outermost exochorion, outer endochorion, transverse layer of pillars with aeropyles, inner endochorion, and the innermost chorionic layer. Interestingly, such features were comparable with those of blow flies [19]. However, no plastron region or median area was detected in *S. dux*.

Morphological information of immature *S. dux* revealed distinct morphological features of larvae. As with most sarcophagid species, larvae possessed posterior spiracles situated within a terminal concavity of the last abdominal segment. Larvae exhibit a light microscopic observation of the cephalopharyngeal skeleton of the first instar displayed apparent anterodorsal process; the anterior end terminally curved downward. The length of the dorsal cornua was slightly longer than the ventral cornua, with the terminal end of the dorsal cornua projecting slightly downward. The dental sclerite was large, attaching the base of the hook part [10]. For the second instar, the dorsal cornua displayed a distinctive feature in having a narrow elongate window, and the length was much longer than the ventral cornua. The terminal end of the dorsal cornua slightly pointed upward. The dental sclerite became small. Regarding the third instar, small dental sclerite was observed. In contrast, the parastomal sclerite was apparent, being slightly curved apically upward. Comparing the third instar of *S. dux* with other forensically important species, although they are morphological similar in appearance and difficulty in identification, our preliminary study using LM revealed that the dorsal spines between the first and second thoracic segments are different from *Boettcherisca nathani* Lopes and *Lioproctia pattoni* (Senior-White). This investigation is on-going research. Besides LM, *S. dux* larvae has been described by SEM, highlighting the important characteristics of the cephalic region (terminal organs, dorsal organs and ventral organs), the ventrally curved mouth hooks, anterior and posterior spiracles [20]. The anterior spiracle located, at the lateral side of the prothorax, shows a single row of 14–17 papillae marginally. The posterior spiracle is D-shaped with an incomplete peritreme. An inner arc is quite pronounced. The distance between both posterior spiracles was narrow, separated by about one third of the spiracle’s width [10]. Such features described for *S. dux* was distinctive with the other medically important flesh fly species, *Liopygia ruficornis* (Fabricius) and *Boettcherisca peregrina* (Robineau-Desvoidy), *B. nathani* and *L. pattoni*. Specifically on the anterior spiracle, the number and arrangement of papillae was morphological different; 10–15 papillae arrange in a single row of *L. ruficornis*; 21–27 papillae arrange in two rows in *B. nathani*; 24–26 papillae arrange in one or two rows in *B. peregrina*; 20–28 papillae papillae arrange in one or two rows in *L. pattoni* (unpublished data). This information was mandatory in using identification of these medically important sarcophagids.

**Figure 8:**
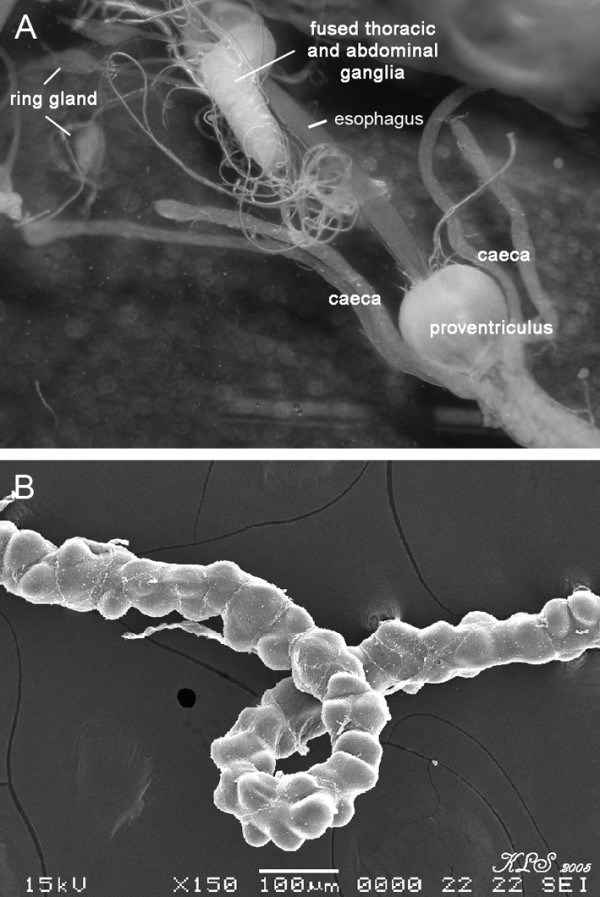
**Micrographs of third instar** ***S. dux*** **. A**: Anterior end displaying esophagus, proventriculus (cardia), gastric caecae. A pair of ring gland and fused thoracic and abdominal ganglia are apparent. **B**: Malpighian tubules.

Information pertaining to internal organs of sarcophagids was limited. Our dissection of the alimentary canal of mature third instar larvae revealed the large crop, a pair of tubular salivary glands, straight tube esophagus connected to bulb-like structure proventriculus (cardia). Posterior of the cardia is four tubular structure of gastric caecae (Figure 8A). Malpighian tubules form long chained of long tubule (Figure 8B) (unpublished data). Such features of these alimentary canal (e.g., crop, salivary glands, esophagus, proventriculus, gastric caecae, Malpighian tubules) are morphologically similar to those third instar of *C. megacephala*[21].

**Figure 9:**
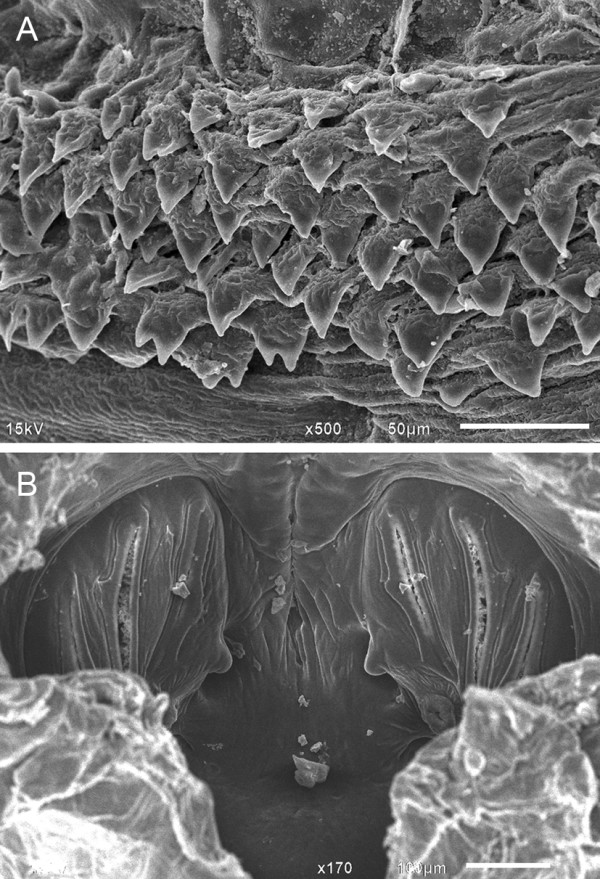
**Scanning electron micrographs of puparium** ***S. dux*** **. A**: Broad and triangular intersegmental spines between the prothorax and mesothorax. **B**: Posterior spiracle.

Puparia of *S. dux* are in the form of coarctate - cylindrical in shape - and composed of the hardened larval integument of the last third instar. They are measured 9.9 ± 0.3 mm in length and 3.8 ± 0.2 mm in width. Under SEM, the intersegmental spines between the prothorax and mesothorax are broad and triangular (Figure 9A), which is resemble *L. ruficornis*; but different from those of *B. nathani* and *L. pattoni* (unpublished data). Based on such distinction, this feature was one of the characteristic used to differentiate among puparia of these four flesh fly species. No pupal respiratory horn was observed dorsolaterally in the first abdominal segment, and this was concordance with those observed in *L. ruficornis*, *B. nathani* and *L. pattoni*. Each posterior spiracular disc appears D shaped, with a pronounced medial projection and three vertically oriented long, narrow spiracular slits (Figure 9B) [22].

### Molecular analysis

Molecular-level studies have addressed taxonomic status of organisms to distinguish morphologically similar species or even genera, including flesh flies. For example, molecular analysis using mitochondrial cytochrome oxidase gene subunits I and II (COI and COII) sequences of 17 Malaysian species of forensic importance successfully clustered into distinct clades and grouped accordingly: *peregrina, albiceps, dux, pattoni, princeps* and *ruficornis. S. dux* was classified as Clade C of the *dux*-group, comprising *S. dux* and *S. brevicornis* Ho [4]. Identification of forensically important sarcophagids from Egypt and China [*S. dux, Sarcophaga argyrostoma* (Robineau-Desvoidy), *Sarcophaga albiceps* (Meigen) and *Wohlfahrtia nuba* (Wiedemann)] was potentially assessed by using partial mitochondrial cytochrome oxidase I and II genes [23]. In four Chinese sarcophagid species including *S. dux*, the 289-bp fragment of the mitochondrial 16S rDNA gene and the 278-bp fragment of the mitochondrial COI gene of DNA method can be used as a supplemental means for morphological method in identification [24]. The genetic characterization of three flesh fly species has been compared [*S. dux*, *S. argyrostoma* (Robineau-Desvoidy) and *L. ruficornis*] using allozyme and RAPD-PCR markers, which indicated a very close relationship between these species [25].

Specifically for *S. dux*, molecular analysis using the sequencing of a 658-bp ‘barcode’ fragment of the mitochondrial COI gene to accurately identify adult sarcophagids from the Australian east coast demonstrated the intraspecific variation within the nonmonophyletic species of *S. dux*, as depicted from the NJ tree, indicating two distinct species which is portrayed graphically by separate clusters [26]. In addition, genetic variability of population has been analysed by electrophoretic profiles using allozymes at five enzyme loci [(Malic enzyme (ME), Acid phosphatase (ACPH), Alkaline phosphatase (APH), Lactate dehydrogenase (LDH) and Xanthine dehydrogenase (XDH)]. All enzymes were found to be encoded at a single locus. These profiles revealed that ME and XDH were monomorphic, whereas APH, LDH and ACPH displayed polymorphism for two electromorphs and three electrophoretic phenotypes, suggesting a low values of genetic variability with the deficiency of heterozygotes in two loci [27].

### Bionomics

Although sarcophagids are commonly viviparous, depositing larvae directly onto a breeding medium; however, some species occasionally lay eggs. Examples of these species observed in laboratory conditions included *S. dux, L. ruficornis, B. nathani* and *L. pattoni*[10, 28]. Habitats in Thailand colonized by *S. dux* are amphibiodotic (larviposition on both faeces and carrion), similar to *L. ruficornis* and *S. annandalei* Senior-White [29].

Developmental rate of fly larvae is mandatory to be applied to estimate the postmortem interval (PMI_min_) in forensic investigations. For flies including *S. dux*, larval development (larviposition until pupariation) varied depending on time of year and location of study. In Thailand, development from the newly hatched larvae to pupariation of this species required 72 h during the summer months (March–June) with temperatures ranging between 27.1–29.8°C. In rainy season and winter, ~96 h were required [13]. In Guam 7 days at 29.5°C were required [30], while in South Africa (=*S. exuberans*) 8.2-10.2 days at 25°C were needed [31]. In Malaysia, the average developmental time of the second instar, third instar, post-feeding, pupa took 19 h, 40.5 h, 73 h and 91 h, respectively, based on fluctuation temperature of 28.9 ± 1.2°C and 64 ± 10% RH [32]. Research from Saudi Arabia indicated that development from first instar to adult emergence was 51.8, 33.0, 25.0, 16.4 and 15.1 d when reared at 16, 20, 24, 28, 32 and 36°C, respectively [33].

Although *S. dux* is prevails in a widespread regions, its bionomic was surprisingly rare. This fly species is a synanthropic [29]. Research conducted in northeast Thailand indicated that adult *S. dux* were collected in the customized reconstructable funnel fly traps baited with 250 g of 1-day tainted beef located in the garbage piles and school cafeteria and not in rice paddy fields [3]. Based on this record, *S. dux* is likely to be endemic. In Thailand, adults were captured from both the flower of the *Bulbophyllum putidum* (Teijsm. & Binn.) plant, *Tectona grandis* L. and *Dimocarpus longan* Lour. fruit [29]. Adults *S. dux* were also associated with cow dung in Malaysia [34], and had some attraction to human excrement [35]. And, this species has been collected at altitudes of 2,000 m above sea level in Nepal [6], indicating the well-adapted to high altitude environments. Our survey assessment using an automatic trap in various land-used types (forested landscape, orchard environments and palm plantations) in Chiang Mai, northern Thailand, is ongoing conducted. This automatic trap, invented by one of authors (K. Sukontason), would help to clarify not only seasonal distribution, but also daily activity of this species and other medically important flies (e.g., blow flies, muscids). Yet, the knowledge of this view is still very limited. Scientific knowledge pertaining to this aspect is required in order to understand bionomics, distribution and richness for different local spots, thereby allowing to be used in forensic investigations, if specimens of *S. dux* are found in the human corpses.

### Myiasis

There is a little published research on the myiasis in human caused by sarcophagids in Thailand. Myiasis cases caused by flesh flies may remain underreported, only that caused by *L. ruficornis* were recorded [36]. Although information regarding *S. dux* as a producing-myiasis agent in humans was very rare in the literature, we recently found that this fly cause aural myiasis in 5-day-old infant in Thailand. Identification of this species was accomplished through morphological characters of male genitalia of adult reared from the larvae recovered (unpublished data). This finding demonstrated rising of myiasis aspect caused by *S. dux*. Such recent case called for attention to the need for protection against flies closely associated with humans including this species. Regarding myiasis in animals, although records was also limited, with skin lesion of camels in India being documented [9]. It has been reported as parasitic on locust and cause bovine tissue myiasis [35].

### Forensic entomology

Few human death investigations have recovered *S. dux*. However, a partial explanation for the lack of *S. dux* associated with human remains is the difficulty in identifying larvae or adults. However, this fly is recognized as forensic important in other regions the Iberian Peninsula [37] and in Switzerland where adult *S. dux* were found associating with human corpse [1].

Flies are the primary invertebrates to colonize animal carcasses and/or bait both on the ground and on the high-rise building. Investigation in Nan province of northern Thailand, *S. dux* adults were collected from domestic pig carcasses (*Sus scrofa* L.), in both suburban and forested areas during all seasons (summer, rainy season and winter) [38]. A study in Malaysia also found this species to be quite active larvae collected on chicken livers located on a rooftop (101.58 m from the ground) [39]. Immature and adult *S. dux* were also collected from rabbit carcasses during winter in Guangzhou, China [40].

## Conclusions

Summarizing, despite *S. dux* prevails in many part of the world and is well recognized in medical view, information on this species being relatively limited. Despite its significance in public health seems likely nonentity, the role as myiasis-producing agent and forensic entomology increasingly brighten. There is a need to enhance study on various bionomic of this species. This would include developmental data in various temperature conditions, behavior, flight activity, seasonal prevalence and/or any research related to be applied in forensic entomology. Although such an investigation will require time, resources and expertise, efforts should be either maintained or initiated since it will not only be beneficial in Thailand, but several countries where this species exists.

## Methods

To examine the morphology of *S. dux*, light microscopy (LM) and scanning electron microscopy (SEM) techniques was employed to observe the important characteristics each stage in its life cycle, except the egg which was published elsewhere [18]. To determine the anatomical feature of immature stages, focusing on the alimentary canal of third instar, mature larvae were dissected and examined under light microscopy, based on the procedure previously described [41]. To examine the morphology of adult, we focused on the genitalia, both ovipositor and male genitalia, which the latter is the most important characteristic used to differentiate flesh fly species. Flies were dissected to obtain their ovipositor or male genitalia by cutting their abdominal segments between 3^rd^ and 4^th^ segments on the clean glass slide using a sharp blade. The terminal tip of the posterior end was then transferring into a well containing 10% potassium hydroxide mixed with 95% ethanol for 3 days. The specimens were rinsed with distilled water before dissection in a centrical-well paraffin plate containing 0.85% NaCl. To dissect ovipositor, two fine long needles were used to cut the sternite. Once the ovipositor stretched, this part was transferred into a well containing 70% alcohol, 80% alcohol and 90% alcohol, each placement for 30 min. The specimens were transferred onto a clean glass slide. Alcohol was removed from the specimens using filter paper, and then xylene was added onto the specimens. A few drop of Permount was added onto the stretched ovipositor, and then cover with the coverslip. The ovipositor was then observed under light microsope and photographs were made using a digital camera (Pentax™, Japan). Illustration of ovipositor and male genitalia were performed using Adobe Illustrator CS4.

Regarding the SEM, puparia and adult of *S. dux* were processed. Puparia were firstly cleaned by washing process that they were placed in gauze and wrapped, and placed in a plastic cup, which was suspended in a beaker (500 ml) contained distilled water. The beaker which bore a magnetic stirrer bar at the bottom was placed onto a hotplate (Barnstead/Thermolyne, Model: SP46920-26, USA) for 3 h. The cleaned puparia were allowed to dry in small petri dish left at room temperature for 7 days, then attached to double-stick tape on an aluminum stub, coated with gold in sputter-coating apparatus, and viewed under a JEOL-JSM6610LV scanning electron microscope. However, to view the posterior spiracle clearly, puparia were placed in 10% KOH for 1–2 days before being cut using sharp blade in the middle of the seventh abdominal segment. The cut part containing posterior spiracle was then attached to double-stick tape on aluminum stub. With respect to adult, flies were processed for SEM observation, as previous described [42].
